# Magnetic CoFe_2_O_4_ nanoparticles supported on graphene oxide (CoFe_2_O_4_/GO) with high catalytic activity for peroxymonosulfate activation and degradation of rhodamine B

**DOI:** 10.1039/c7ra09949e

**Published:** 2018-01-03

**Authors:** Rida Tabit, Othmane Amadine, Younes Essamlali, Karim Dânoun, Abdallah Rhihil, Mohamed Zahouily

**Affiliations:** Laboratoire de Matériaux, Catalyse & Valorisation des Ressources Naturelles, URAC 24, Faculté des Sciences et Techniques, Université Hassan II Casablanca B.P. 146 20650 Morocco m.zahouily@mascir.com +212 661416359; MAScIR Foundation, VARENA Center, Rabat Design Rue Mohamed El Jazouli Madinat Al Irfane 10100 Rabat Morocco

## Abstract

Herein, we report the preparation of magnetic CoFe_2_O_4_ nanoparticles and CoFe_2_O_4_/graphene oxide (GO) hybrids and evaluate their catalytic activity as heterogeneous peroxymonosulfate (PMS) activators for the decomposition of rhodamine B. The surface morphologies and structures of both CoFe_2_O_4_ nanoparticles and CoFe_2_O_4_/GO hybrids were investigated by powder X-ray diffraction (XRD), scanning electron microscopy (SEM), energy-dispersive X-ray spectroscopy (EDS), Fourier transform infrared spectroscopy (FTIR) and nitrogen adsorption–desorption isotherms. The magnetic properties of the samples were assessed using a SQUID magnetometer at 298 K. Catalytic oxidation experiments demonstrated that CoFe_2_O_4_/GO hybrids exhibited much better catalytic activity than CoFe_2_O_4_ nanoparticles or CoFe_2_O_4_/reduced graphene oxide (rGO) hybrids, suggesting that GO plays an important role in CoFe_2_O_4_/GO hybrids in the decomposition of rhodamine B. The influence of various reaction conditions such as temperature, concentration of PMS, pH and decomposition time of rhodamine B over the CoFe_2_O_4_/GO catalyst were investigated and optimized. The rhodamine B degradation process was found to fit a pseudo-first order kinetics model. The catalyst could be easily separated from the reaction mixture by applying an external magnet. In particular, the as-prepared CoFe_2_O_4_/GO hybrid exhibited good reusability and stability in successive degradation experiments in PMS solution.

## Abstract 2

Herein, we report the preparation of magnetic CoFe_2_O_4_ nanoparticles and CoFe_2_O_4_/graphene oxide (GO) hybrids and evaluate their catalytic activity as heterogeneous peroxymonosulfate (PMS) activators for the decomposition of rhodamine B.

## Graphical abstract

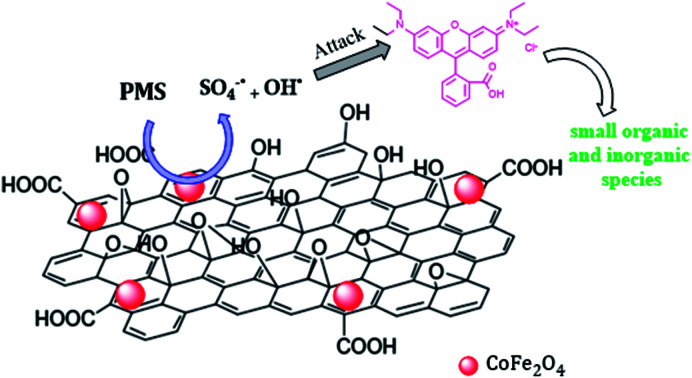

## 1. Introduction

The dye industry discharges large amounts of industrial wastewater and hence, it is one of the major sources of organic pollutants. Nowadays, there are more than 100 000 dyes belonging to various chemical classes with an annual production of 7.105 tons.^1^ It is estimated that 10–15% of initial quantities are lost during dyeing procedures, which are discharged without prior treatment to the effluent; this contaminates groundwater and is toxic to humans and animals. Most of these compounds are chemically stable and have a complicated constitution, which makes them resistant to photo- and biological degradation. The Rhodamine (RhB) dye is one of fresh peach of synthetic dyes that is widely used as a colorant in the manufacturing of textiles and foodstuffs. It has been medically proven that rhodamine dye is harmful and toxic to humans and animals, and causes irritation of the skin, eyes and respiratory tract.^2^ Due to its high toxicity and negative effects on public health, various physical, chemical and biological approaches have been extensively explored and investigated for the removal of organic dyes from wastewater including adsorption, coagulation, biological degradation and filtration processes as well as chemical oxidation by hydro chlorite and Fenton methods.^3^ However, these methods suffer from different drawbacks that are primarily associated with the cost-intensive production of oxidants, instability during long reaction times, short lifetime and pH adjustments.

In recent years, sulfate radical-based oxidation processes have received much attention^4^ for its efficient degradation of organic contaminants. The sulfate radical (SO˙^4−^) generated from peroxymonosulfate, as an alternative to the hydroxyl radical (OH˙), is a strong oxidant with a high redox potential. It can react with many organic contaminants to yield a degradation performance similar to that expected for the hydroxyl radical (OH˙). The activation processes of PMS can be achieved using heat, ultraviolet irradiation, transition metals, or metal oxides,^5–7^ which are similar to the cases involving hydrogen peroxide. Recently, the Co^2+^ ion coupled with a PMS system for the degradation of organic contaminants has attracted tremendous interest since it exhibits better efficiencies than the Fenton reaction.^8,9^ Despite the advantages of this homogeneous activation process, the application of this method in water treatment is limited due to pollution caused by the high solubility and significant toxicity of transition metal ions and the complex recovery of metal ions from the reaction medium. One of the most favorable ways to overcome these drawbacks is through activation of PMS *via* heterogeneous systems,^8–10^ such as Co/activated carbon,^11^ Co/carbon aerogel,^12^ Co/carbon xerogels,^13^ Co_3_O_4_,^14–16^ Co-exchanged zeolites,^17,18^ Co/SBA-15,^19–21^Co/mesoporous MnO_2_,^22^ and Co/MCM-41.^23^ Among the various heterogeneous catalysts, magnetic cobalt ferrites (CoFe_2_O_4_), belonging to the family of spinel-type ferrites, have attracted extensive attention due to their large surface area, high catalytic activity, stable crystalline structure and particularly their easy separation from the reaction system by utilizing magnetic fields derived from their ferromagnetic properties.^24–26^ The cobalt ferrites prepared through conventional methods generally consist of highly agglomerated particles with low specific area, which reduces their catalytic performance.^27^ To solve this agglomeration problem, several synthetic routes have been developed. Among the developed approaches, dispersing agglomerated particles onto the various supports was found to be an effective method to enhance the catalytic activity of CoFe_2_O_4_.^28,29^ The enhancement of the catalytic activity of the CoFe_2_O_4_-supported catalyst was due to the synergic effect between CoFe_2_O_4_ and the support. The role of the support was not negligible in this case. Recently, immobilization of CoFe_2_O_4_ nanoparticles onto exfoliated graphite oxide has been the subject of intense research due to the excellent properties and functionalities of the resultant hybrid material as well as its wide spectrum of applications, which include catalysis, biomedicine and decontamination of waste water.^30–33^

In the present study, we report a facile approach for preparing magnetic CoFe_2_O_4_ nanoparticles, CoFe_2_O_4_/reduced graphene oxide (rGO) and CoFe_2_O_4_/graphene oxide (GO) and their catalytic performance toward activating PMS for the removal of rhodamine B. The physicochemical properties of all samples were characterized by various techniques, such as nitrogen adsorption–desorption, SEM, XRD and FTIR. The catalytic activities of all prepared samples were investigated in terms of the reaction kinetics, reaction temperature, concentration of RhB and catalytic stability.

## 2. Experimental

### 2.1. Materials

**Scheme 1:**
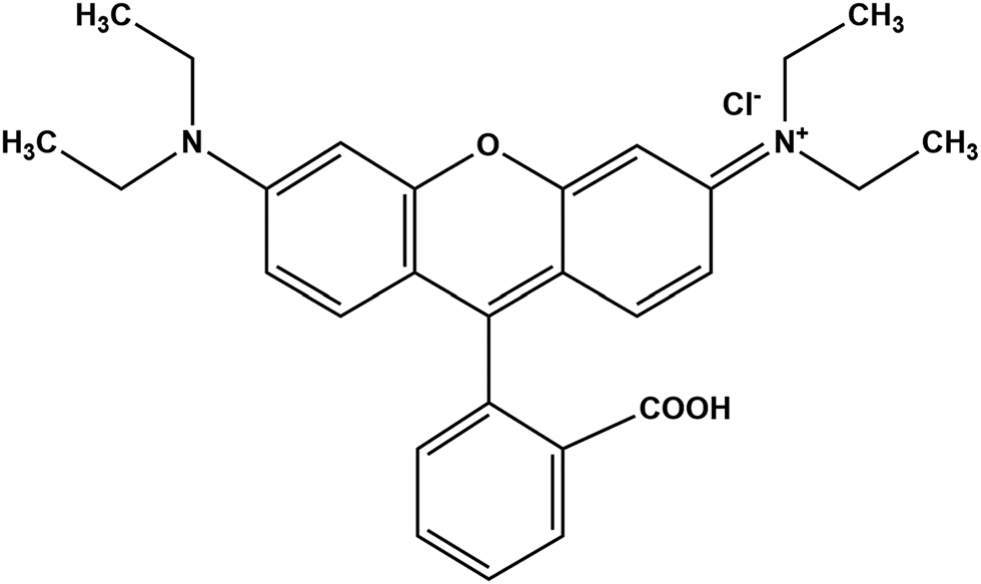
Structure of rhodamine B.

High purity rhodamine B (C_28_H_31_CIN_2_O), oxone (KHSO_4_·K_2_SO_4_·KHSO_5_), iron chloride hexahydrate (FeCl_3_·6H_2_O), cobalt chloride hexahydrate (CoCl_2_·6H_2_O), sodium hydroxide (NaOH), graphite, sodium nitrate (NaNO_3_), sulfuric acid H_2_SO_4_ (98% w/w), potassium permanganate KMnO_4_, hydrogen peroxide H_2_O_2_ (30% w/w) and C_2_H_6_O were purchased from Aldrich chemical company. All the reagents were used without further purification. Water used in all experiments was deionized. The molecular structure of rhodamine B is shown in Scheme 1.

### 2.2. Preparation of GO and CoFe_2_O_4_/GO

Graphene oxide (GO) was prepared from natural graphite *via* the modified Hummers method.^34^ In the preparation of CoFe_2_O_4_–GO, 0.16 g of GO was first dispersed in water and sonicated for 30 min to produce a homogeneous brown dispersion of graphene oxide nanosheets. Separately, 2.7 g of FeCl_3_·6H_2_O and 1.19 g of CoCl_2_·6H_2_O were dissolved in 10 mL of distilled water under vigorous stirring. The bimetallic Fe–Co solution was then added to the GO dispersion and the resultant mixture was further stirred for 1 h. Then, 25 mL of an aqueous solution of NaOH (3 mol L^−1^) was added dropwise to the above dispersion under vigorously stirring. Later, the dispersion was heated in a sand bath for 1 h at 100 °C. Finally, the resultant precipitate was magnetically separated, washed with water and ethanol until the pH was neutral (pH = 7) and dried at 60 °C for 24 h. For comparison, CoFe_2_O_4_ NPs were also prepared according to the above procedure.

### 2.3. Characterization

XRD measurements were recorded on a Bruker AXS D-8 diffractometer using Cu-Kα radiation in Bragg–Brentano geometry (*θ*–2*θ*). All samples were also characterized by Fourier-transform infrared spectroscopy in the range of 4000–400 cm^−1^ using an ABB Bomem FTLA 2000 spectrometer with 16 cm^−1^ resolution. SEM and STEM micrographs were obtained on a Tecnai G2 microscope at 120 kV. The elemental composition of the CoFe_2_O_4_/GO nanocomposite was confirmed from energy dispersive X-ray analysis (EDAX). The surface areas of the prepared materials were measured using the Brunauer–Emmett–Teller (BET) method on a 3Flex automatic analyzer. Prior to N_2_ sorption, all samples were degassed at 250 °C for 8 h. The magnetic properties of CoFe_2_O_4_ nanoparticles and CoFe_2_O_4_/GO nanocomposite were investigated in a MPMS-XL-7AC superconducting quantum interference device (SQUID) magnetometer. The magnetic measurements were performed from −15 000 to 15 000 Oe at room temperature. Total organic carbon (TOC) was determined by the Shimadzu TOC-L Series. Cobalt and iron within CoFe_2_O_4_/GO were determined by inductively coupled plasma atomic emission spectroscopy (ICP-AES) from Jab in Yvan and the carbon content was determined by a carbon/sulfur analyser (C.A) using HORIBA EMIA-320V2.

### 2.4. Catalytic test procedure

The catalytic degradation of RhB by CoFe_2_O_4_ and CoFe_2_O_4_/GO catalysts with oxone was performed in a 50 mL beaker containing 50 mL of RhB solution at room temperature (25 °C). In a typical procedure, 0.005 g of oxone was first added to RhB solution under constant stirring. Then, 0.010 g of catalyst was added to start the reaction. At a given time interval, a predetermined amount (2.0 mL) of solution was withdrawn into a vial fitted with a micro-filter (45 μm) for solid catalyst removal. The concentrations of RhB were determined by monitoring the decrease in absorbance at the maximum wavelength (554 nm) with UV-vis spectroscopy.

The degradation efficiency was calculated according to the following equation:
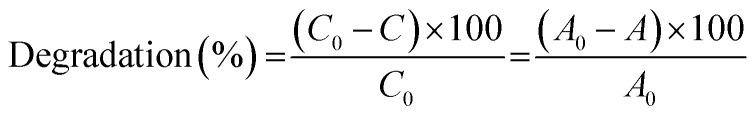
where, *C*_0_ and *C* are the initial and final concentrations of RhB, respectively, and *A*_0_ and *A* represent the initial and the final absorbance of RhB at 554 nm, respectively.

## 3. Results and discussion

**Fig. 1:**
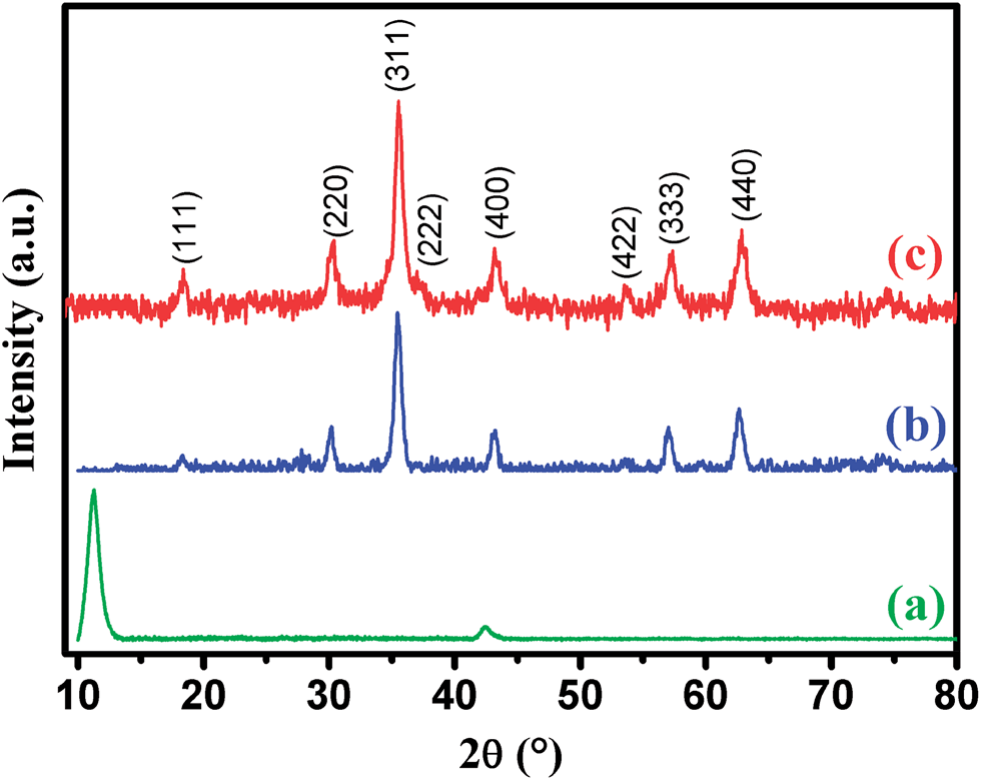
XRD patterns of graphene oxide (a), CoFe_2_O_4_ (b) and CoFe_2_O_4_/GO (c).

XRD was employed to analyze the crystalline phases of as-prepared GO, CoFe_2_O_4_ and CoFe_2_O_4_/GO samples (Fig. 1). The XRD patterns (Fig. 1a) confirmed the successful oxidation of natural graphite to graphite oxide, which exhibits a strong diffraction peak at 2*θ* = 10.28, corresponding to the (001) inter-planar spacing of 0.87 nm, which is much larger than the *d*-spacing of graphite (0.34 nm). It is evident that CoFe_2_O_4_ and CoFe_2_O_4_/GO exhibit similar XRD patterns. The diffraction peaks for the two samples at 2*θ* = 30.3, 35.6, 43.3, 53.6, 57.1 and 62.8 are consistent with the (220), (311), (400), (422), (333) and (440) reflections, respectively, of the cubic spinel-type structure of CoFe_2_O_4_ (JCPDS 75-0033). It should be noted that typical diffraction peaks of GO could not be detected in the XRD pattern of CoFe_2_O_4_/GO, suggesting the destruction of the regular layered structure of GO due to the crystal growth of CoFe_2_O_4_ between its inter-layers. The average crystallite size of CoFe_2_O_4_ and CoFe_2_O_4_/GO, estimated from the Debye–Scherrer formula, are 10.37 and 11.06 nm, respectively.

**Fig. 2:**
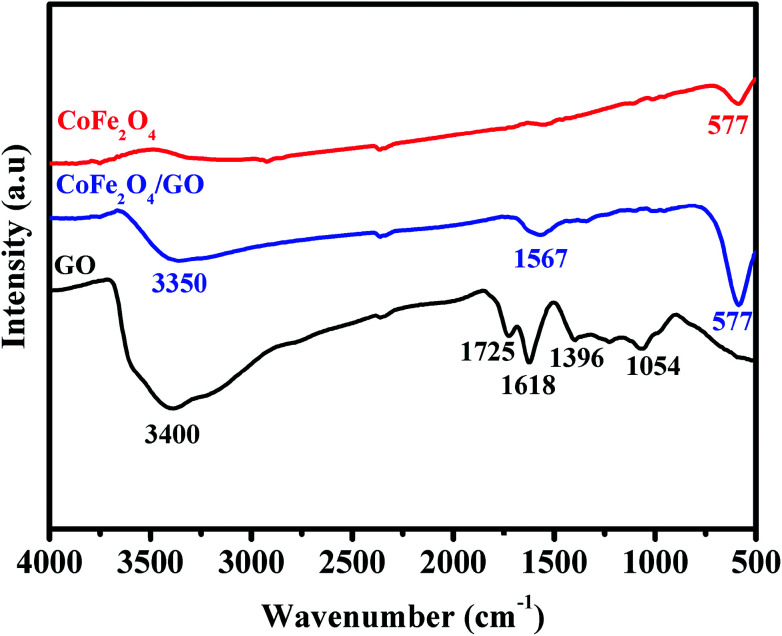
FT-IR spectra of GO, CoFe_2_O_4_ and CoFe_2_O_4_/GO.

The FT-IR spectra of GO, CoFe_2_O_4_ and CoFe_2_O_4_/GO are shown in Fig. 2. As illustrated in Fig. 2, several characteristic bands of the functional groups of GO can be observed. The two peaks located at 1725 and 1618 cm^−1^ are assigned to the anti-symmetric and symmetric stretching vibrations of carboxylic groups (COO^−^). The absorption peaks at 1035 cm^−1^ is attributed to the stretching vibrations of alkoxy C–O from the oxygen-containing functional groups such as carbonyl, carboxylic and epoxy groups.^35^ Indeed, the FTIR spectrum of CoFe_2_O_4_ shows a peak at 577 cm^−1^, which is ascribed to the Co–O and Fe–O vibrations. It is worth noting that in the spectrum of CoFe_2_O_4_/GO, the peaks at 1618 cm^−1^, corresponding to the functional groups of COO in GO, shifts to 1567 cm^−1^.

**Fig. 3:**
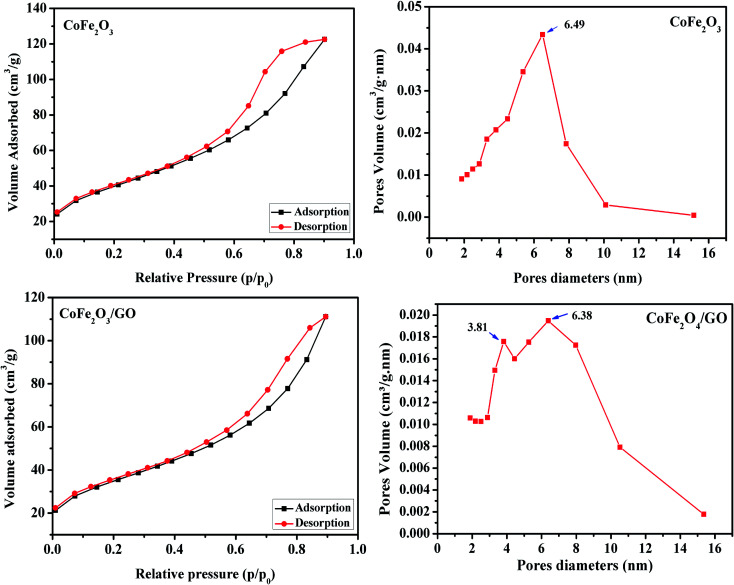
Nitrogen adsorption/desorption isotherms and BJH pore size distribution of CoFe_2_O_4_ and CoFe_2_O_4_/GO.

The calculated textural parameters of CoFe_2_O_4_ and CoFe_2_O_4_/GO are summarized in Table 1. From the data in Table 1, it can be seen that the BET surface area of CoFe_2_O_4_ is 124 m^2^ g^−1^ and its pore volume is 0.1662 cm^3^ g^−1^, while the surface area of the CoFe_2_O_4_/GO is 142 m^2^ g^−1^ and its pore volume is 0.1868 cm^3^ g^−1^. The addition of GO nanosheets should be the responsible for this increase in the surface area of CoFe_2_O_4_/GO compared to that of CoFe_2_O_4_. The nitrogen sorption isotherm of CoFe_2_O_4_, as shown in Fig. 3, is of type III with a distinct hysteresis loop of type H2 in the relative pressure range of 0.5 and extending almost to 1, which is characteristic of mesoporous textural porosity. The corresponding pore size distribution curve indicates that CoFe_2_O_4_ exhibited a pore size distribution in the range of 2 to 10 nm with a central value of 6.5 nm, corresponding to mesoporous materials. As for CoFe_2_O_4_/GO, there is a slight difference as the nitrogen sorption isotherms of CoFe_2_O_4_/GO are of the type III (Fig. 3) and the hysteresis loop is of type H3 according to the IUPAC classification. The corresponding pore size distribution curve indicates that CoFe_2_O_4_/GO has a centralized pore size distribution within two areas toward 4 and 6 nm, which confirm the existence of textural mesopores.

**Table 1:** Lattice parameter, crystallite size, BET surface area and pore volume of the CoFe_2_O_4_ and CoFe_2_O_4_/GO samples

Samples	Lattice parameter *a* (Å)	Cell volume (Å^3^)	Crystallite size^a^/nm	*S*_BET_ b/m^2^ g^−1^	*V*_meso_ b/mL g^−1^	*D*_meso_ b/nm
CoFe_2_O_4_	8.399	592.50	10.37	124	0.1662	6.5
CoFe_2_O_4_/GO	8.354	583.02	11.06	142	0.1868	4.6

a Calculated by the Debye–Scherer equation.

b From nitrogen sorption analysis.

**Fig. 4:**
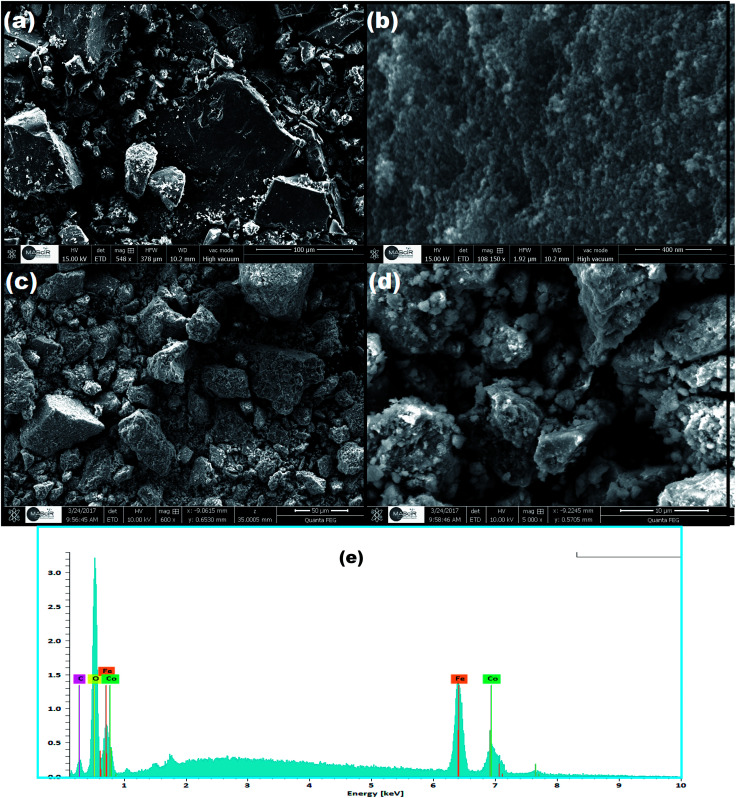
SEM images of (a, b) CoFe_2_O_4_ and (c, d) CoFe_2_O_4_/GO, (e) EDX of CoFe_2_O_4_/GO.

The surface morphology and elemental composition of the as-prepared CoFe_2_O_4_ and CoFe_2_O_4_/GO were investigated by SEM and EDX. From the SEM images, as shown in Fig. 4, it could be observed that CoFe_2_O_4_ exhibits a heterogeneous microstructure that consisted of crystallites of various sizes. It should be noted that particles of CoFe_2_O_4_ are strongly agglomerated as shown in the FE-SEM images in (Fig. 4b), which can be attributed to the powerful inherent magnetic interaction of CoFe_2_O_4_ magnetic particles. Furthermore, as shown in Fig. 4c and d, when CoFe_2_O_4_ was supported on the surface of GO nanosheets, the agglomeration phenomenon reduced, suggesting that GO can prevent the aggregation of the CoFe_2_O_4_ nanoparticles. Moreover, energy dispersive X-ray (EDX) analyses were recorded and are shown Fig. 4e. The EDX analysis confirmed the elemental composition of the CoFe_2_O_4_–GO material, which primarily consists of C, Co, Fe, and O. The chemical composition of the as-synthesized products was further analyzed by inductively coupled plasma atomic emission spectroscopy (ICP-AES). Table 2 shows the elemental composition of CoFe_2_O_4_/GO measured by EDX, ICP-AES and C.A. The stoichiometric elemental composition for Co, Fe, and O is 22.79, 44.44, and 4.32%, respectively. In all samples, Co and Fe are in the ratio of 1 : 2, which further confirm the structure of CoFe_2_O_4_/GO.

**Table 2:** Elemental composition of CoFe_2_O_4_/GO measured by EDX, ICP-AES and C.A

Element	Weight (%)		
	EDX	ICP-AES	C.A
Co	22.15	22.79	—
Fe	45.94	44.44	—
C	4.24	—	4.32
O	37.65	—	—

**Fig. 5:**
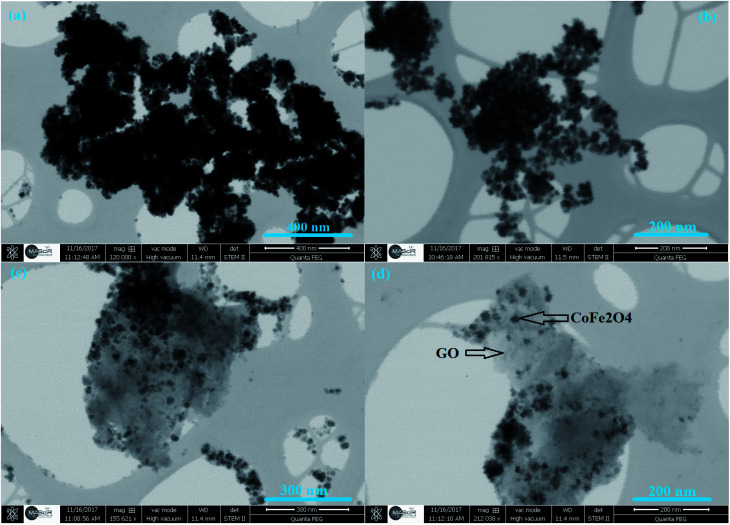
STEM images of CoFe_2_O_4_ (a, b) and CoFe_2_O_4_/GO (c, d).

The morphology of GO and CoFe_2_O_4_/GO was characterized by STEM. From Fig. 5(a and b), it can be observed that CoFe_2_O_4_ NPs prepared in the absence of GO show spherical particles with severe aggregation due to the magnetic dipolar interaction among the magnetite NPs. In comparison, uniform CoFe_2_O_4_ NPs are deposited and well-dispersed on the basal planes of graphene (Fig. 5(c and d)). In addition, we noticed that CoFe_2_O_4_ NPs were still tightly anchored on the surface of GO even after sample preparation (mechanical stirring and sonication) for STEM analysis, suggesting a strong interaction between CoFe_2_O_4_ NPs and GO. Moreover, the graphene sheets distributed between the CoFe_2_O_4_ NP can prevent the aggregation of CoFe_2_O_4_ NP to a certain extent, which can be of great benefit to reactions.

**Fig. 6:**
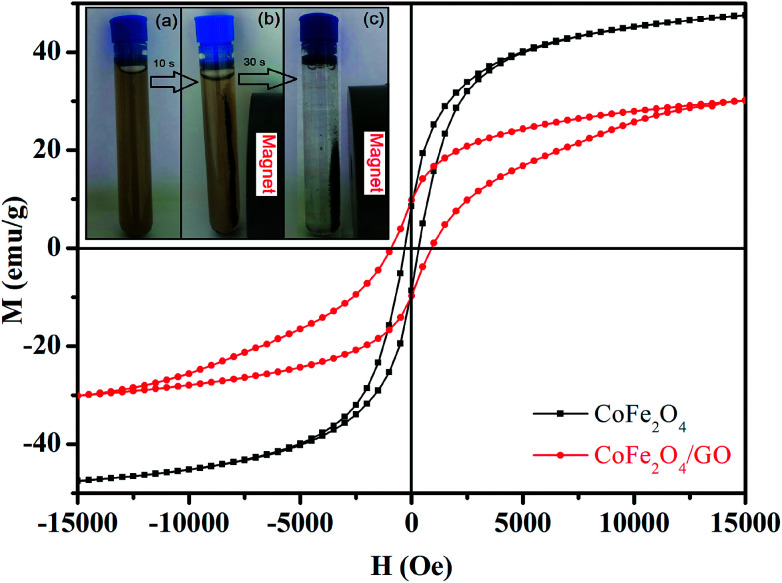
Magnetization curves of CoFe_2_O_4_ and CoFe_2_O_4_/GO. The inset shows the photographs of the separation processes of CoFe_2_O_4_/GO: (a) without external magnetic field, and (b, c) with external magnetic field.

The magnetic properties of CoFe_2_O_4_ and CoFe_2_O_4_/GO were investigated by SQUID from −15 000 to 15 000 Oe at room temperature as shown in Fig. 6. The saturation magnetization (Ms) of CoFe_2_O_4_ is close to 47.33 emu g^−1^, which is higher than that corresponding to the CoFe_2_O_4_/GO material (30.15 emu g^−1^). This increase in Ms value could be due to (i) the existence of the GO nanosheets, (ii) the surface defect of CoFe_2_O_4_ crystallites and (iii) the strong interfacial interaction between CoFe_2_O_4_ nanoparticles and GO surfaces. Moreover, the field-dependent magnetization curves of CoFe_2_O_4_ and CoFe_2_O_4_/GO show non-negligible remanence (Mr) and coercivity (Hc), indicating paramagnetic behavior of the two samples at room temperature (Table 3). Furthermore, CoFe_2_O_4_/GO hybrids can be easily separated from the reaction by applying an external magnetic field. The CoFe_2_O_4_/GO catalyst can be dispersed in deionized water to form a stable brown homogenous suspension before magnetic separation (inset of Fig. 6a). However, when a magnet was placed close to the reaction vessel, it could be observed that the synthesized samples were rapidly attracted, and a nearly colorless solution was obtained (inset of Fig. 6(b and c)).

**Table 3:** The values of saturation magnetization (Ms), remanent magnetization (Mr) and coercivity (Hc) of CoFe_2_O_4_ and CoFe_2_O_4_/GO, extracted from Fig. 5

Sample	Ms (emu g^−1^)	Mr (emu g^−1^)	Hc (Oe)
CoFe_2_O_4_	47.33	8.55	348.53
CoFe_2_O_4_/GO	30.15	9.78	897.16

**Fig. 7:**
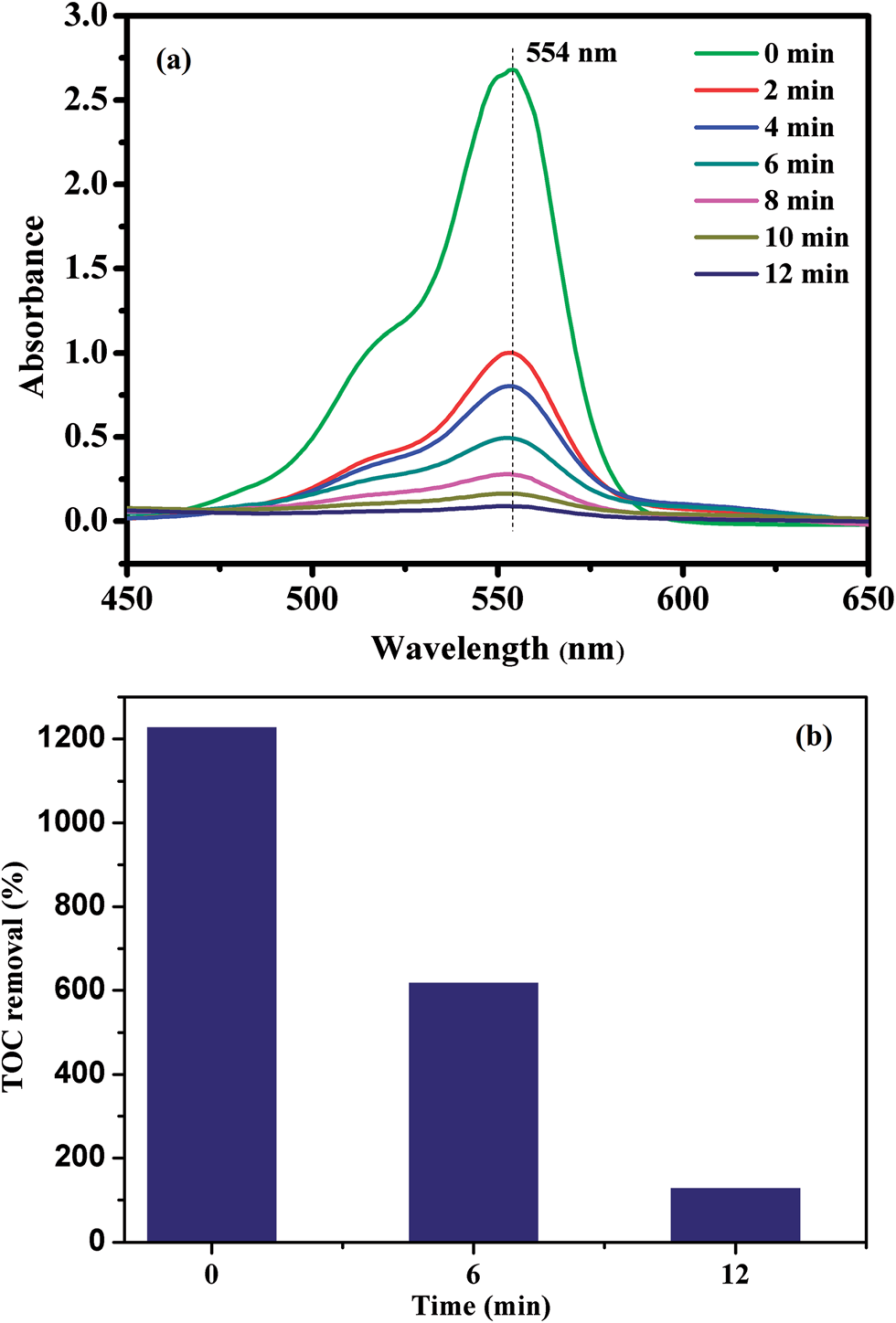
UV-vis spectra of RhB solution at different times using CoFe_2_O_4_/GO as a catalyst (a); the TOC removal efficiency of RhB using CoFe_2_O_4_/GO as a catalyst.

To evaluate the catalytic performance of our materials, the degradation of RhB in the presence of the PMS was selected as a model catalytic reaction. The degradation kinetics of RhB in aqueous solution was studied by monitoring the decrease of its absorption peak at 554 nm in the UV-vis spectra with time as exemplified in Fig. 7a. No significant shift in the absorption peak (*λ*_max_ = 554 nm) was observed before 12 min except for a quick reduction in the absorbance at 554 nm. Since the absorbance at 554 nm was primarily attributed to RhB remaining in the solution under our experimental conditions, the linear relationship between the concentration of the residual RhB and its absorbance at 554 nm was further verified by the standard calibration curve (*R*^2^ = 0.9970), and the corresponding absorbance was then converted to the RhB concentration in the solution used for kinetic analysis. To further investigate the degradation of RhB, total organic carbon (TOC), which has been widely used to evaluate the degree of mineralization of organic species, was measured in the RhB degradation process by the CoFe_2_O_4_/GO/PMS system as shown in Fig. 7b. The TOC removal efficiency of RhB reached 89.34% after 12 min in the presence of the CoFe_2_O_4_/GO/PMS system, which confirms that RhB could be mineralized to residual organic molecules by the as-prepared samples.

**Fig. 8:**
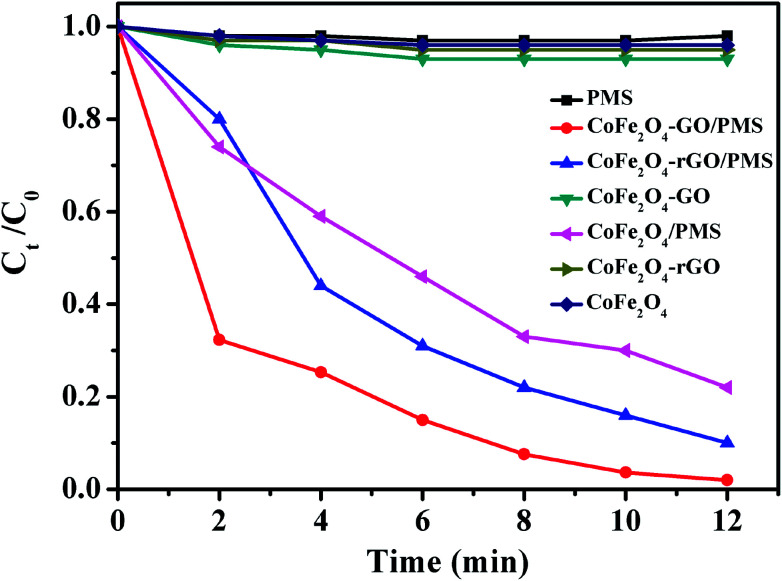
RhB degradation by various catalytic PMS systems. Conditions: [RhB]_0_ = 0.03 mmol L^−1^, [PMS] = 0.10 mg L^−1^, amount of catalyst = 10 mg, temperature = 25 °C.

As shown in Fig. 8, no RhB degradation was observed *via* PMS oxidation alone. Similarly, CoFe_2_O_4_, CoFe_2_O_4_/rGO and CoFe_2_O_4_/GO cannot catalyze RhB degradation in the absence of PMS, which reveals that the contribution from simple physical adsorption is negligible in this case. Moreover, in the absence of a catalyst, the concentration of RhB remained unchanged over time, suggesting that it is difficult for the degradation of RhB to proceed without a catalyst. Therefore, the degradation of RhB is very sensitive to the presence of both PMS and catalyst in the reaction system. The catalytic performances of CoFe_2_O_4_, CoFe_2_O_4_/rGO and CoFe_2_O_4_/GO for the degradation of RhB with PMS were clearly different in the three samples and the degradation efficiencies were 78, 90 and 98%, respectively. Moreover, it can be clearly observed that the degradation rate of RhB over CoFe_2_O_4_/GO was much faster than that corresponding to CoFe_2_O_4_/rGO and CoFe_2_O_4_ and it took around 12 min for complete removal of RhB. This superior catalytic activity of CoFe_2_O_4_/GO could be related to the electronic structure and the presence of functional hydroxyl groups in GO, which could be involved in the degradation mechanism, thus enhancing the catalytic activity of the CoFe_2_O_4_/GO catalyst for the degradation of RhB. As compared with CoFe_2_O_4_, GO can offer an environment to prevent aggregation of CoFe_2_O_4_ nanoparticles and also a higher surface area (142 m^2^ g^−1^) (124 m^2^ g^−1^), which can provide more active sites for catalytic degradation of RhB.

**Fig. 9:**
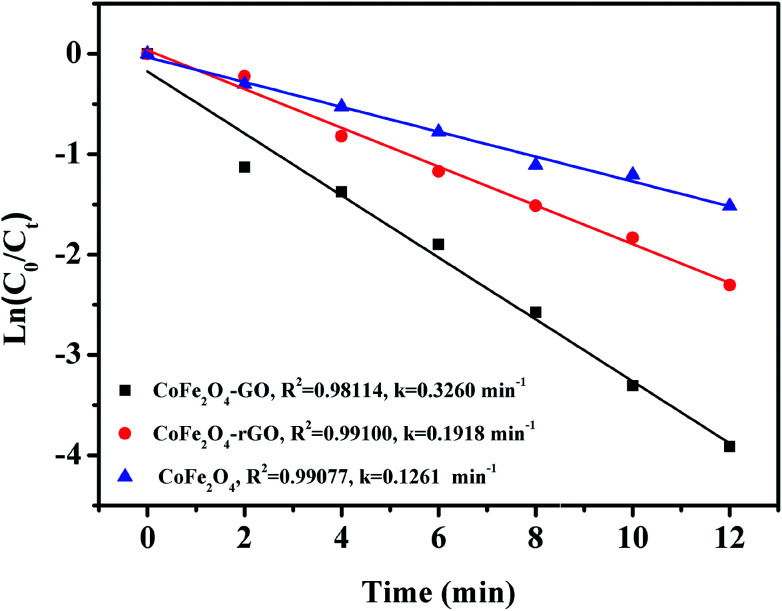
Linear kinetic fitting plots of ln(*C*_0_/*C*_*t*_) = *f*(*t*) based on a first-order kinetic model.

Fig. 9 shows the linear kinetic fitting plots of ln(*C*_0_/*C*_*t*_) = *f*(*t*) for RhB photodegradation, in which *C*_0_ and *C*_*t*_ are the initial concentration of RhB and its concentration at time *t*, respectively. In general, the degradation of organic dyes obeys a pseudo-first order kinetics model. As shown in Fig. 9, RhB degradation by CoFe_2_O_4_, CoFe_2_O_4_/GO and CoFe_2_O_4_/rGO can follow a pseudo-first order kinetics model. Furthermore, the constant rate values indicate that CoFe_2_O_4_/GO possesses a better rate constant (0.3260 min^−1^) than CoFe_2_O_4_/rGO (0.1918 min^−1^) and CoFe_2_O_4_ (0.1261 min^−1^), suggesting that graphene oxide plays a significant role in the enhancement of PMS catalytic degradation of RhB. The strong interfacial interaction between graphene oxide and CoFe_2_O_4_ generates a synergistic function of large surface area and improves the electron transport ability and chemical reaction sites.

**Fig. 10:**
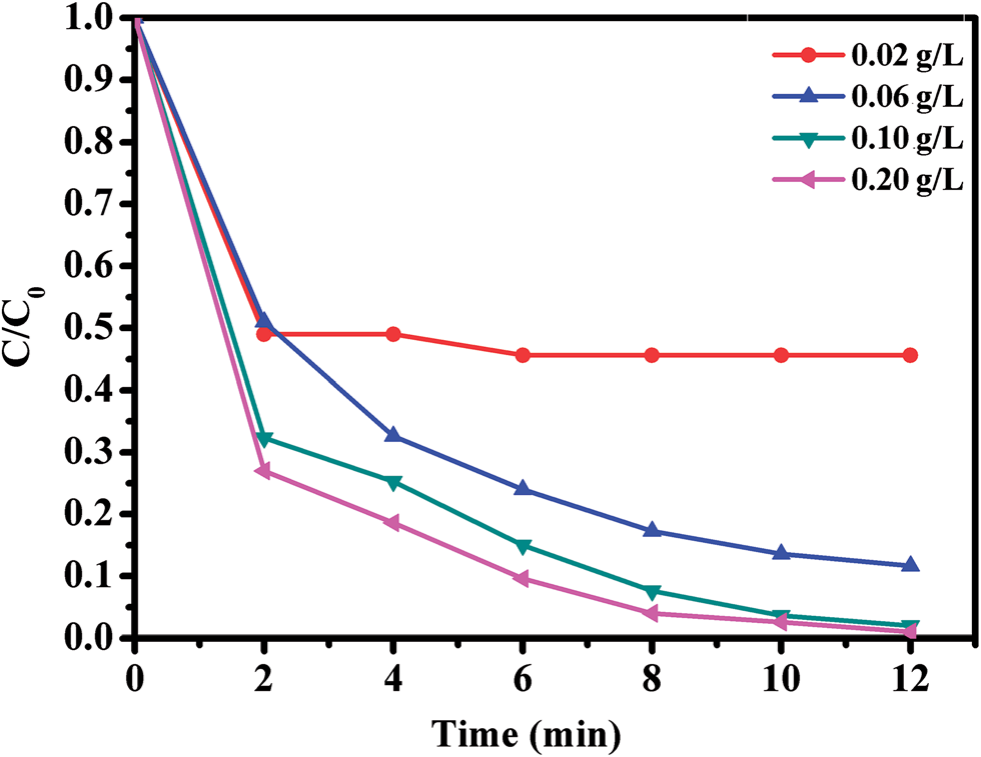
Effect of oxone concentration on RhB degradation over CoFe_2_O_4_/GO. Reaction conditions: [RhB] = 0.03 mmol L^−1^, amount of catalyst = 10 mg, *T* = 25 °C.

The effect of oxone concentration on RhB degradation was studied and the results are presented in Fig. 10. It is worth mentioning that increasing oxone concentration from 0.02 to 0.20 g L^−1^ led to a faster and more efficient degradation of RhB from 54.4 to 99%. Meanwhile, the kinetic rate constant also increased from 0.0654 to 0.1918 min^−1^. These results could be explained by the high concentration of free sulfate radicals formed at higher oxone concentration, which result in an increase in the rate of RhB degradation. Therefore, the optimal oxone concentration was about 0.1 g L^−1^ for the degradation of RhB on the CoFe_2_O_4_/GO catalyst.

**Fig. 11:**
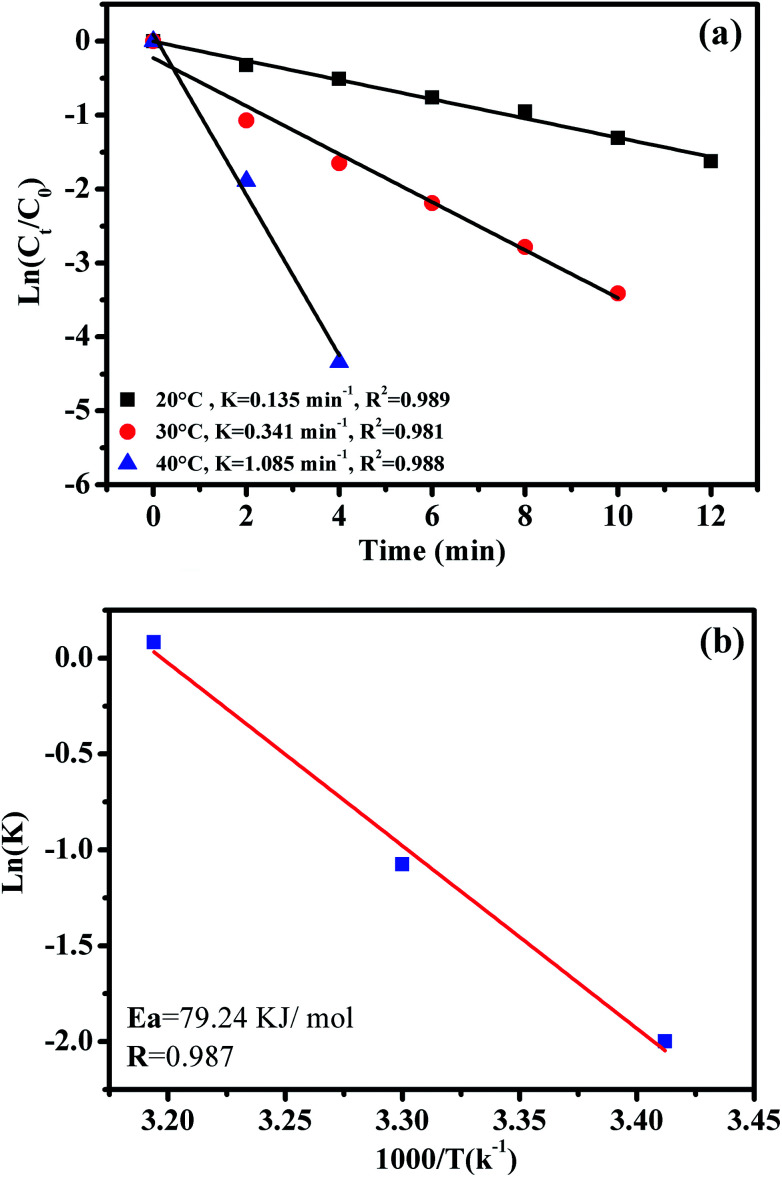
The effect of reaction temperature on RhB degradation over the CoFe_2_O_4_/GO catalyst (a); the Arrhenius plot with linear regression (b); reaction conditions: [RhB] = 0.03 mmol L^−1^, amount of catalyst = 10 mg and 0.1 g L^−1^ of oxone at different temperatures.

The effect of reaction temperature on RhB degradation was also investigated by varying the reaction temperatures from 20 to 30 to 40 °C; the experimental findings are presented in Fig. 11. It can be observed that the degradation rate of RhB increases from 0.135 to 1.085 min^−1^ with increasing temperature from 20 to 40 °C. These results could be explained by the high concentrations of free sulfate radicals generated at high temperature.

To further evaluate the effect of the CoFe_2_O_4_/GO nanocatalyst on the process kinetics, important parameters associated with the energetic aspects of the reaction, such as activation energy, (*E*_a_) play a crucial role. The activation energy (*E*_a_) of the degradation of RhB over the CoFe_2_O_4_/GO catalyst was evaluated by plotting ln(*k*) *versus* 1000/*T* according to the Arrhenius equation of ln(*k*) = ln(*A*) −*E*_a_/*RT*, where *k* is the rate constant, *R* is the universal gas constant (8.314 J mol^−1^ K) and *A* is the pre-exponential. The *E*_a_ value of 79.24 kJ mol^−1^ was obtained from the slope of the fitted equation by linear regression (*R*^2^ = 0.987). This *E*_a_ value is comparable to those of the highest active heterogeneous catalysts ever reported, *e.g.*, Co/active carbon (59.7 kJ mol^−1^),^11^ Co_3_O_4_/SiO_2_ (61.7–75.5 kJ mol^−1^)^36^ and Co/ZSM-5, (69.7 kJ mol^−1^),^17^ which indicates that CoFe_2_O_4_–GO can be considered a promising heterogeneous catalyst for the PMS oxidation process.

**Fig. 12:**
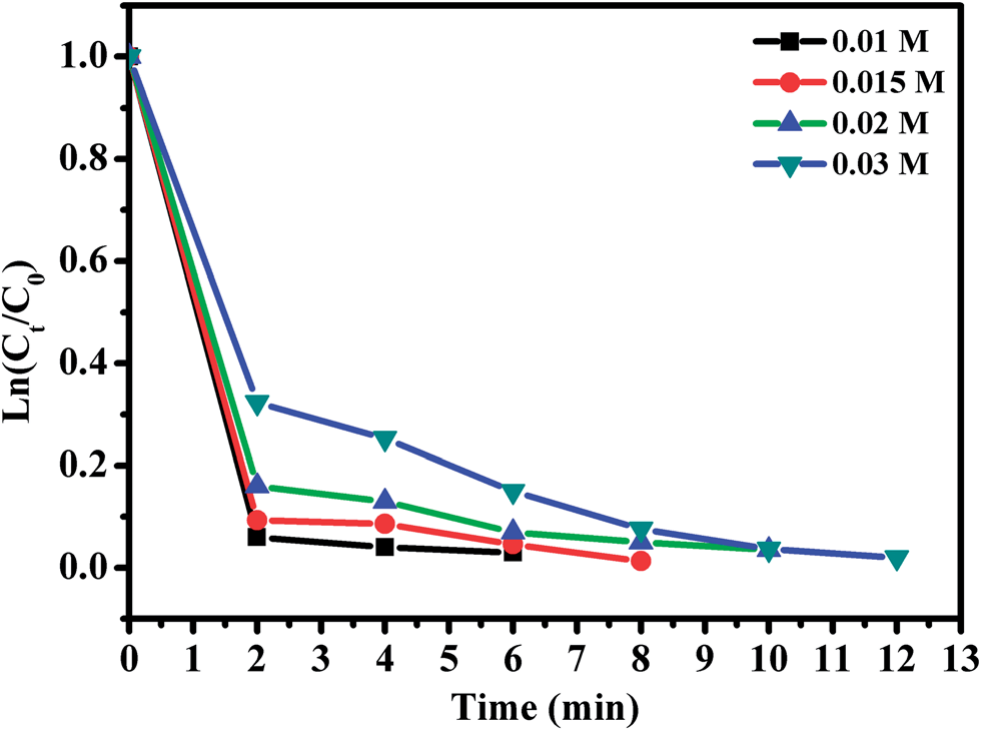
Effect of RhB concentration. Reaction conditions: [PMS] = 0.1 mmol L^−1^; amount of catalyst = 10 mg, *T* = 25 °C.

The effect of RhB concentration was studied by varying the concentration of RhB and the experimental findings are presented in Fig. 12. Upon decreasing RhB concentration from 0.01 to 0.03 mmol L^−1^, the degradation rate of RhB over CoFe_2_O_4_/GO increased significantly. Indeed, RhB was almost completely removed within 6 min at the RhB concentration of 0.01 mmol L^−1^, while it was removed within 12 min at the concentration of 0.03 mmol L^−1^.

**Fig. 13:**
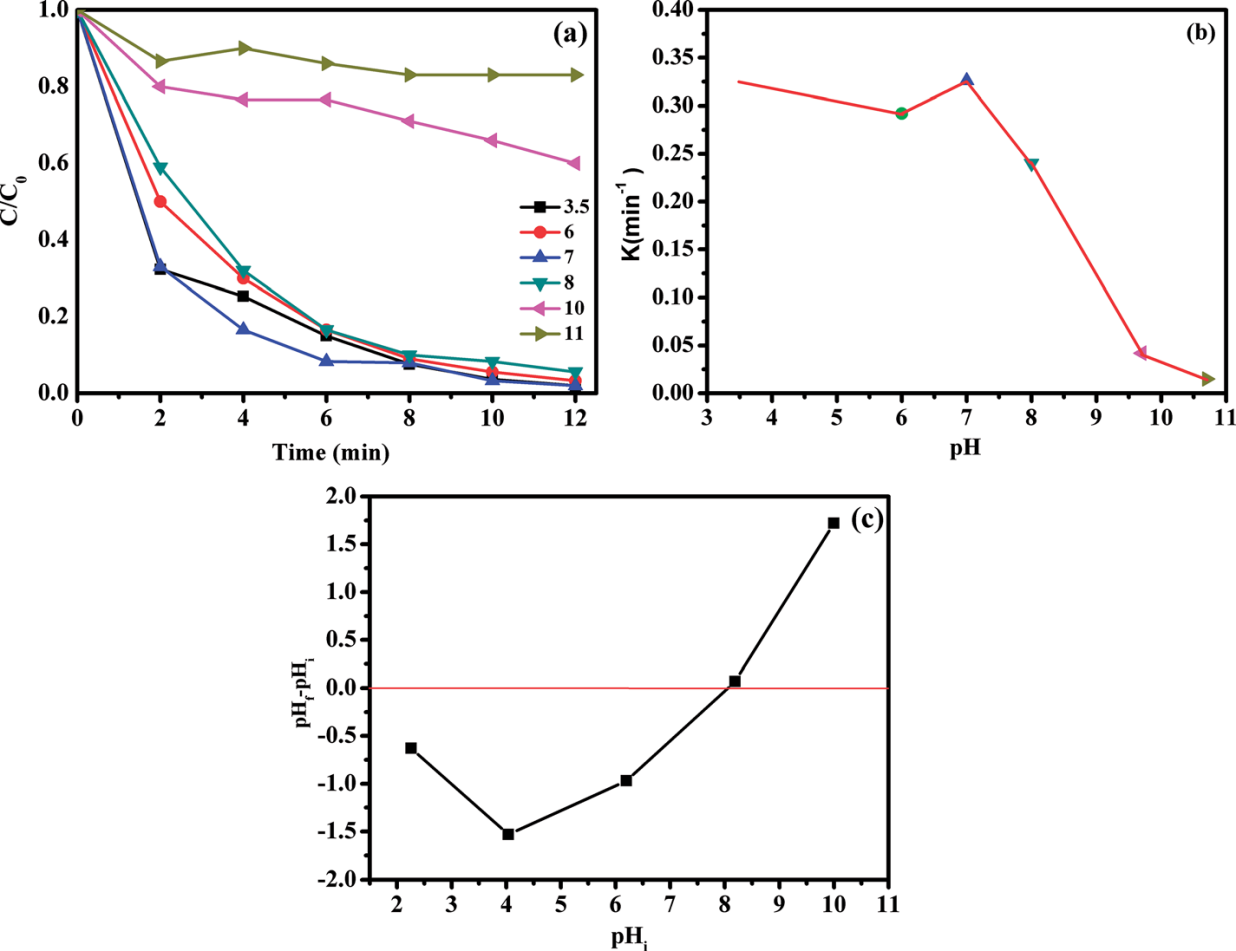
Effect of pH on RhB degradation over the CoFe_2_O_4_/GO catalyst (a, b); point zero charge of CoFe_2_O_4_/GO. Reaction conditions: [RhB] = 0.03 mmol L^−1^, [PMS] = 0.10 mmol L^−1^, amount of catalyst = 10 mg, *T* = 25 °C.

The influence of initial pH values on RhB degradation over the CoFe_2_O_4_/GO catalyst was explored by adjusting the solution pH to 3.5, 6, 7, 8, 10 and 11. As shown in Fig. 13, the initial pH has a significant influence on the degradation efficiency of RhB. Based on the results obtained, we observed that the RhB degradation conducted at an initial pH of 3.5 and 7.0 was faster (decolorization efficiency of 98% at about 12 min) due to the electrostatic attraction between the negative charge of the CoFe_2_O_4_/GO catalyst at low pH (Fig. 13c) and the positive charge of RhB. It is also noteworthy that there was no obvious impact on the RhB degradation when the initial pH value changed in the range of 3.5–7. Moreover, at high initial pH values of 10.0 and 11.0, the decolorization rates were 40% and 17%, respectively, which can be explained by the deprotonation of the carboxyl group of RhB and the transformation of the cationic form of RhB into zwitterionic form.

**Fig. 14:**
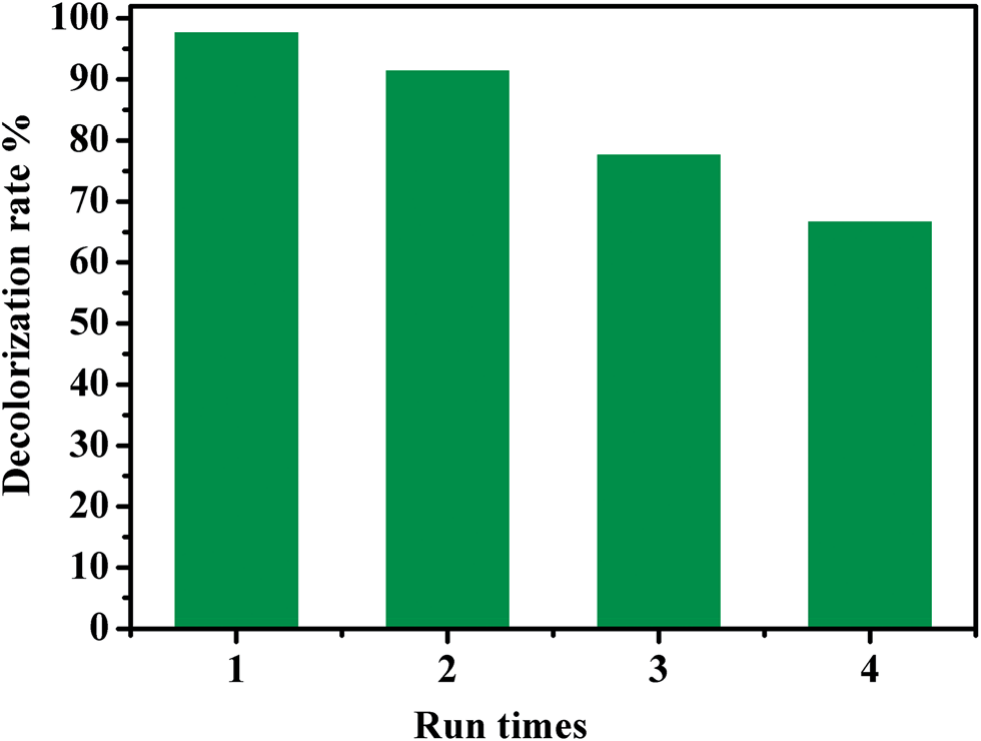
Reuse performance of the CoFe_2_O_4_/GO catalyst in RhB degradation. Reaction conditions: [RhB] = 0.03 mmol L^−1^, [PMS] = 0.10 mmol L^−1^, amount of catalyst = 10 mg, *T* = 25 °C.

The recyclability of a catalyst is advantageous for its commercialization and industrialization. To explore the reusability of our catalytic system, CoFe_2_O_4_/GO nanocomposite was employed as a recyclable catalyst in the degradation of RhB by PMS over four cycles. After each cycle, the catalyst was easily separated by an external magnet and washed successively by water and dichloromethane. As shown in Fig. 14, it was found that the catalytic performance of the recovered catalyst remained nearly the same for the second successive run. Although the catalytic activity slightly diminished, 67% of decolorization rate was still achieved in the fourth run, indicating that CoFe_2_O_4_/GO nanocatalysts exhibited good recyclability. Metal leaching was studied by ICP-AES analysis of the CoFe_2_O_4_/GO catalyst after the reaction. The Co and Fe concentrations in the catalyst was 20.96% and 39.51%, respectively, after one catalytic cycle of RhB degradation, which confirms negligible metal leaching.

## 4. Conclusion

In summary, magnetic CoFe_2_O_4_ and CoFe_2_O_4_/GO catalysts were successfully prepared *via* a facile approach. The physico-chemical properties of these materials were evaluated by FT-IR, XRD, SEM, and BET. These catalysts showed potential capability for catalytic degradation of rhodamine B using PMS as an oxidant. Catalyst screening revealed that CoFe_2_O_4_/GO exhibited superior catalytic activity for the removal of RhB when compared with CoFe_2_O_4_ and CoFe_2_O_4_/rGO. Then, we studied the effect of several parameters on RhB degradation. It was found that the degradation rate was dependent on pH, temperature, the concentration of oxone and the initial concentration of RhB. Furthermore, this catalyst can be easily separated by an external magnet and reused.

## Conflicts of interest

There are no conflicts to declare.

## Supplementary Material
